# Spontaneously shrinking lung mass due to *Mycobacterium avium* mimicking lung cancer

**DOI:** 10.1002/ccr3.5006

**Published:** 2021-11-06

**Authors:** Takeshi Kinjo, Kohei Uechi, Wakaki Kami, Jiro Fujita

**Affiliations:** ^1^ Department of Infectious, Respiratory and Digestive Medicine Graduate School of Medicine University of the Ryukyus Nakagami‐gun Japan; ^2^ Division of Clinical Laboratory and Blood Transfusion Ryukyu University Hospital Nakagami‐gun Japan

**Keywords:** calcification, mass, *Mycobacterium avium*, nontuberculous mycobacteria, solitary pulmonary nodule

## Abstract

NTM‐SPN is often indistinguishable from malignancy. Although surgical resection is sometimes chosen for the diagnosis and treatment, the mass in this case shrank spontaneously. Careful observation is required to avoid unnecessary interventions.

## Graphical abstract

Nontuberculous mycobacterial disease presenting as a solitary pulmonary nodule (NTM‐SPN) is often indistinguishable from malignancy. Although surgical resection is sometimes chosen for the diagnosis and treatment, the mass in this case shrank spontaneously. Careful observation is required to avoid unnecessary interventions.

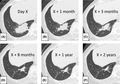

## 1 CLINICAL IMAGES

We present a case of nontuberculous mycobacterial pulmonary disease presenting as a solitary mass mimicking lung cancer. Although surgical resection is sometimes chosen for diagnosis and treatment purposes, this mass shrank spontaneously. This case had an uncommon clinical course and demonstrates the need for careful observation to avoid unnecessary interventions.

**FIGURE 1:**
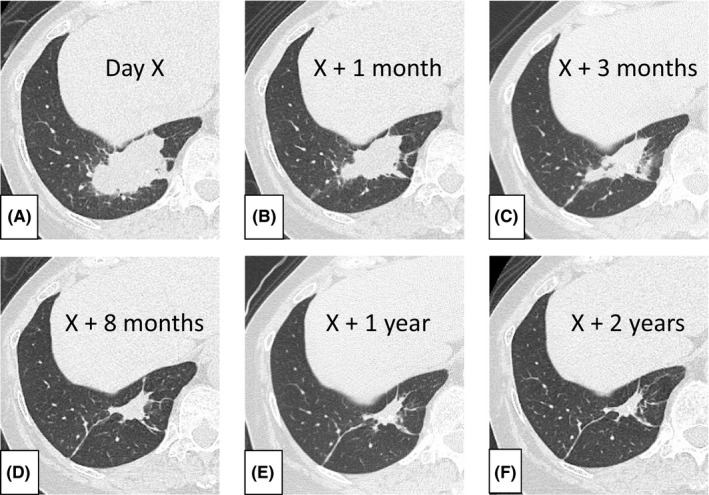
A solitary mass (6 × 3 cm) with spiculation and internal calcification was observed in the right inferior lobe of the lung (A). Thereafter, the mass spontaneously shrank without any treatment (B–F)

A 63‐year‐old woman with lymphomatoid papulosis was referred to the respiratory department for evaluation of an incidental pulmonary mass observed on chest computed tomography. She had no symptoms except for the cutaneous lesion. The mass, measuring 6 × 3 cm, was in the right inferior lobe of the lung and had spiculation and internal calcification (Figure 1A). A bronchoscopy revealed no evidence of malignancy; however, 2 weeks later, a liquid culture for acid‐fast bacilli from bronchial lavage fluid yielded a positive result. *Mycobacterium avium* was subsequently identified by mass spectrometry. Eventually, the mass shrank spontaneously with accompanying scarring (Figure 1B‐F), and follow‐ups were terminated 2 years later.

Nontuberculous mycobacterial disease presenting as a solitary pulmonary nodule (NTM‐SPN) is rare and often indistinguishable from malignancy. Spiculation, which is a representative radiological finding of lung cancer, is also observed in 21%–42% of NTM‐SPNs.^1^, ^2^ Thus, diagnosing NTM‐SPNs is often difficult. In a review of the literature, Kobashi, et al.^1^ found that 11/12 NTM‐SPN cases required surgical resection for diagnosis. Although 22 NTM‐SPN cases with a mass larger than 3 cm have been reported, there are no previous reports of cases presenting with a spontaneously shrinking mass. Although the precise mechanism is unknown, we speculated that immune response, as explained in self‐healed tuberculosis, might be involved in the spontaneous shrinking phenomenon. The clinical course, in this case, was uncommon, demonstrating that careful observation is required to avoid unnecessary interventions.

## CONFLICT OF INTEREST

The authors declare no conflicts of interest.

## AUTHOR CONTRIBUTIONS

TK, KU, WK, and JF: writing of the draft and approval of the manuscript for submission.

## CONSENT

Written informed consent was obtained from the patient for the publication of this clinical image.

## Data Availability

Data sharing is not applicable to this article as no datasets were generated or analyzed during the current study.
